# Planning and adjustments for the control of reach extent in a virtual environment

**DOI:** 10.1186/1743-0003-10-27

**Published:** 2013-03-02

**Authors:** Jill Campbell Stewart, James Gordon, Carolee J Winstein

**Affiliations:** 1 Division of Biokinesiology and Physical Therapy at the School of Dentistry, University of Southern California, 1540 Alcazar Street, CHP 155, Los Angeles, CA, 90089-9006, USA; 2 Department of Neurology, Keck School of Medicine, University of Southern California, Los Angeles, CA, USA

**Keywords:** Upper extremity, Reaching, Motor planning, Virtual reality

## Abstract

**Background:**

Skilled performance of reach actions includes both anticipatory planning and compensatory adjustments made while moving. The execution of reach actions in a virtual environment (VE) demonstrates similar characteristics to reaches performed in the real-world, however, it is unclear whether the VE itself significantly impacts movement planning or compensatory adjustments. The purpose of this study was to directly compare the use of planning and adjustments to control extent for unconstrained reach actions performed in an immersive VE to those performed in an analogous real-world environment (RWE).

**Methods:**

Five non-disabled adults (29 ± 5 years) reached with the dominant, right arm to six targets presented in two directions (+45°, -45°) and three distances (8, 16, 24 cm) in a VE and an analogous RWE. Position data were sampled at 120 Hz from an electromagnetic marker on the index finger and differentiated to determine velocity and acceleration. The control of reach extent was compared between the two environments (paired *t*-test) as to the use of planning (correlation of peak acceleration with movement distance), compensatory adjustments prior to peak velocity (correlation of time to peak velocity with movement distance), and compensatory adjustments after peak velocity (variance in movement distance accounted for by deterministic statistical model).

**Results:**

Reach movements were relatively fast (<400 msec) and scaled to target distance in both the VE and RWE. Overall, the control of reach extent was similar in all respects between the two environments. In both environments, a hybrid control pattern was observed. That is, individuals utilized a combined strategy that relied on both planning and compensatory adjustments to capture the target. Adjustments to the reach were evident prior to peak velocity through changes in acceleration duration as well as after peak velocity based on target information. The two factor deterministic statistical model (peak velocity, target distance) explained >92% of the variance in movement distance across participants and environments.

**Conclusions:**

The VE did not impact movement planning or subsequent compensatory adjustments for the control of reach extent when directly compared to an analogous RWE. An immersive VE is a valid environment for the study of unconstrained reach actions.

## Background

Virtual reality (VR) is a computer-generated environment that simulates the real-world and provides an interface through which the user can interact with objects and events [1-3]. While VR is an emerging tool for use in the systematic and controlled practice of physical tasks for rehabilitation [1,3], VR can also be used to investigate the principles that govern goal-directed upper extremity (UE) actions such as the control of movement direction and extent [4,5], the role of movement execution noise [6], and the integration of sensory information [7,8]. Yet, it is unclear if the virtual environment (VE) itself has an influence on the motor control principles being investigated. Research suggests that the execution of reach actions in VR have both similar and different characteristics when compared to the execution of reach actions in the real-world [9-12]. It is not known, however, whether the VE impacts the planning of reach actions. A variety of environmental factors have been shown to influence the planning of reach actions such as the compatibility between target presentation space and movement space [13], provision of background visual stimuli [14-16], and mode of target presentation [8,17-21]. Therefore, it is important to understand the impact of a VE on the planning of goal-directed actions to allow for appropriate interpretation of experimental data collected in such an environment.

An immersive VE provides a unique opportunity to study the planning of reach actions. In an immersive display, virtual objects are presented to the user in first-person space; the user reaches to objects displayed directly in front of him similar to everyday, real-world functional tasks (compatibility between target and movement workspace). This differs from experimental task environments where objects are presented on a vertical computer screen but require the user to move in the horizontal space directly in front of him (incompatibility between target and movement workspace). Environments with differences between the target presentation space and movement space present unique sensorimotor transformation requirements that impact motor planning compared with environments were the target and movement space are the same [13]. Therefore, research findings on movement planning obtained in an immersive VE may have a direct impact on the understanding of goal-directed actions in everyday life.

Several features of VR make it attractive for the investigation of the control of motor actions. Three-dimensional (3-D) control of virtual objects allows systematic manipulation of the location and timing of target presentation. For example, sequential presentation of a single target can be provided while other targets are not visible. Manipulation of visual feedback allows control of whether the user sees the moving effector or a representation of the effector; timing and duration of visual information can be altered based on the research question of interest. Virtual object presentation can also be manipulated such that the compatibility between actual movements and visual stimuli is altered allowing the researcher to change the sensorimotor transformation requirements of an experimental task. With an immersive VE, all of these features can be utilized while the user interacts with an environment presented directly in front of him. Several studies have taken advantage of these unique features to investigate the control of reach actions [4-8], however, investigators assumed that there was no effect of the VE on the outcome of interest.

One well-described characterization of planned reach actions is a systematic scaling of reach kinematics to targets that vary in distance [5,22-26]. Non-disabled individuals scale the magnitude of peak velocity to target distance when reaching to targets that vary in distance; they use lower peak velocities for closer targets and higher peak velocities for farther targets [5,22-24]. Individuals can achieve this scaling of peak velocity through two different control patterns: pulse-height control and pulse-width control. Pulse-height control involves varying the magnitude, or height, of peak acceleration [5,27,28]. Since the peak of acceleration occurs early after movement onset before the availability of feedback, this control pattern is thought to be indicative of anticipatory planning. Pulse-width control involves varying the duration, or width, of the acceleration phase during which sensory feedback is available to the movement system [5,29]. A pulse-width control pattern has been hypothesized to act to compensate for any errors in the initial specification of peak acceleration magnitude [30,31]. Additional corrective adjustments are also possible after the time of peak velocity that may assist in the achievement of the desired movement distance [23,32].

Our main research interest lies in understanding the degree to which the control of reach extent is influenced by hemispheric stroke. The first step in pursuing this line of research is to confirm the validity of an immersive VE for the investigation of how individuals plan and adjust reaches to targets that vary in distance. Therefore, the purpose of this study was to directly compare the planning of unconstrained reach actions performed in a VE to those performed in an analogous real-world environment (RWE). Since the two environments did not differ as to the compatibility between target and movement workspace and, therefore, were thought to have similar sensorimotor transformation requirements, we hypothesized that individuals would plan and adjust reach actions in a similar way in the VE and the RWE.

## Methods

### Participants

Six non-disabled adults (mean age 29 ± 5 years) reached with the right arm to designated targets during a single session. All participants were right-hand dominant [33] and had no current or previous neurologic disorder. All participants provided written informed consent prior to participation through a protocol approved by the University of Southern California’s Institutional Review Board. For one participant (S1), errors occurred during data collection in the RWE making the data unusable. Therefore, only data from the five remaining participants with full data sets is reported.

### Experimental task

#### Virtual environment

**Figure 1:**
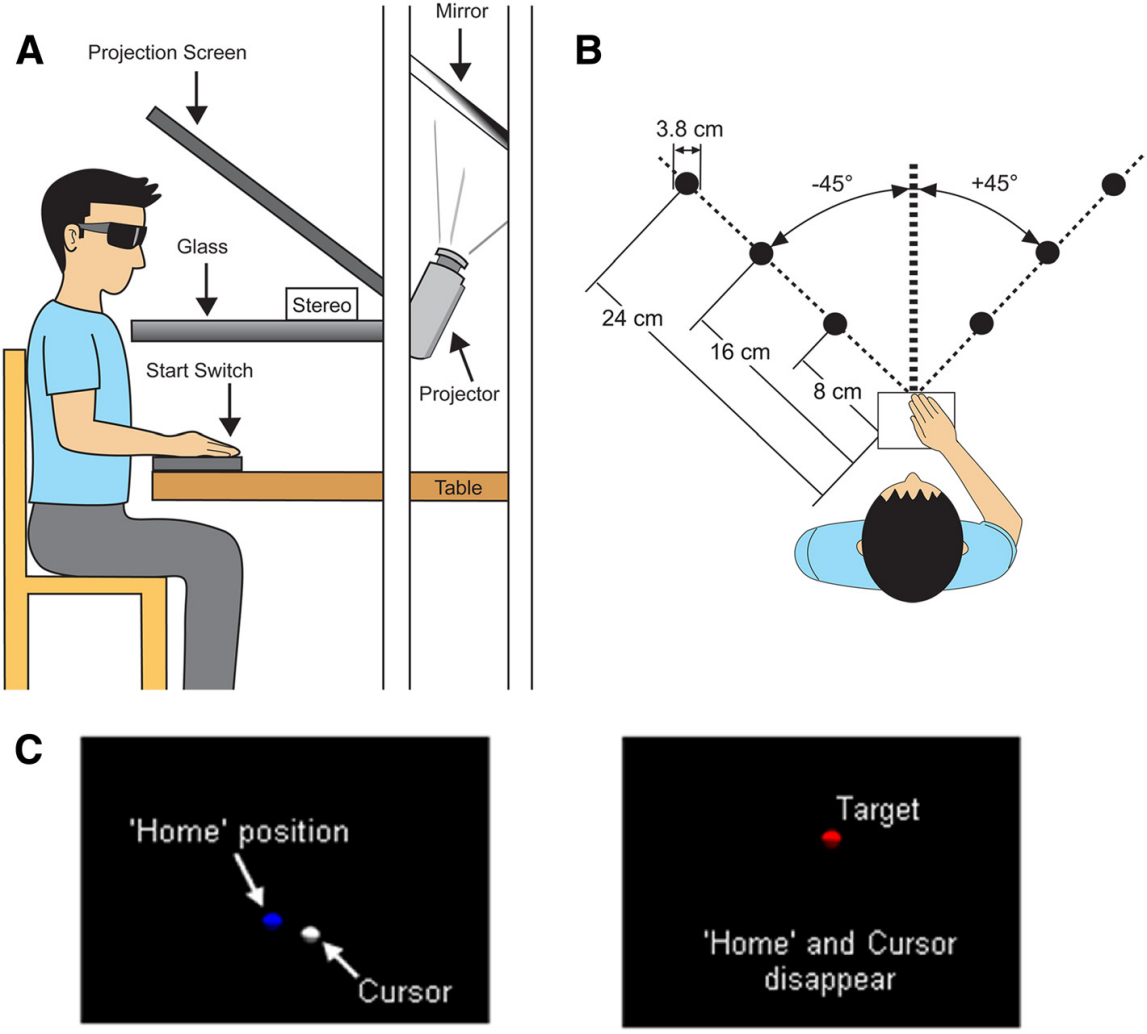
**Experimental task. A**) Side view schematic of participant sitting at virtual display unit. Stereoscopic glasses were worn to allow 3-dimensional view of virtual environment (VE). Virtual objects were sent to the projector and reflected off the mirror into the workspace below the glass. Participants began each trial with the right hand on a physical start switch but ended the reach in free space (above the table). The same table, chair, and start switch were used in the analogous real-world task to keep positioning consistent. **B**) Top down view. Six targets were presented in 2 directions (+45°,-45°) and 3 distances (8, 16, 24 cm). The start switch (open square) aligned with the sternum. The same target locations and physical set up were used in the analogous real-world task. **C**) View of VE. At the start of each trial, participants aligned the Cursor (white sphere which corresponded to finger position) with a Home position (blue sphere). Both the Cursor and the Home position disappeared when the Target appeared (red sphere) eliminating visual feedback while moving.

Targets were presented in an immersive virtual display system (Innovative Sport Training, Inc., Chicago, IL) (Figure 1A). The environment consisted of a black background and colored spheres to indicate finger position and target location (Figure 1C). A single electromagnetic marker placed on the index finger of the right hand acted as the interface for aiming movements. Finger position was represented in the VE as a 2 cm white sphere, or Cursor, that moved in real-time as the participant moved the finger. This same electromagnetic marker was used for collection of position data while reaching. Stereoscopic glasses sampled at 60 hz per eye were worn to allow 3-D visualization of objects. An electromagnetic marker attached to the glasses tracked head movements; the visual display was updated based on changes in head position to allow natural, unrestricted head motion during task completion.

The workspace consisted of six targets (3.8 cm red spheres) presented in two directions (+45°, −45°) and three distances (8, 16, 24 cm) (Figure 1B). At the start of each trial, the participant placed the Cursor onto a Home position represented as a 2.5 cm blue sphere that aligned with a physical start switch. Once the Cursor was on the Home position and maintained for 1 second, the Home position turned yellow as a ready signal. After a variable foreperiod (1.3, 1.6, or 1.9 seconds), the Home position and the Cursor position disappeared and a single red target appeared at which time the participant performed a 3-D reach. The target was visible while reaching but the arm and the finger Cursor were not eliminating on-line visual feedback of movement requiring any feedback based adjustments to be made based on internally derived proprioceptive information [30]. After each trial, visual post-response feedback showing proximity of final finger position to the target was provided. If the Cursor overlapped with the target (error tolerance of 2.9 cm), the target turned green on feedback indicating to the participant that they successfully hit the target on that trial. If the Cursor did not overlap with the target, the target remained red during feedback.

#### Real-world environment

An analogous real-world task (RWE) was created for comparison. The goal was to create an environment that would elicit similar reach movements as the VE, and, therefore, three task conditions were considered important to implement in the RWE. First, all reaches were made in 3-D space. Second, on-line visual feedback of the arm during movement was not provided requiring adjustments to the reach be made based on internally derived proprioceptive information. Third, since haptic feedback was not provided in the VE, reaches ended in open space so that individuals would not alter planning and adjustments to accommodate for endpoint interaction with an object.

For the RWE, the table surface from the VE was moved out of the virtual display and placed in open space. Reach targets were 3.8 cm white spheres placed on top of the flat table in the same two directions (+45°, −45°) and three distances (8, 16, 24 cm) as in the VE (Figure 1B). Use of the same table surface in both environments allowed for standardization of start switch location, target height, and chair height. As in the VE, a single electromagnetic marker on the index finger of the right hand was used to track each aiming movement for data collection.

The participant initiated each trial in the RWE with the index finger positioned on the start switch. After a tone, the researcher verbally indicated the target goal (e.g. right close, left middle, right far, etc.), and the participant visually looked at the target. After a second tone, the participant closed her eyes to eliminate on-line visual feedback of movement and reached to the target. The reach was made above the plane of the target such that when the participant ended the reach the finger was positioned above the goal target in free space (no haptic feedback) similar to the VE. After a third tone, the participant opened her eyes and looked at the finger location in relation to the target for post-response visual feedback before returning to the start switch for the next trial.

### Experimental procedure

All participants reached with the dominant, right arm in the VE first followed by the RWE during a single data collection session. The instructions for reaching in both environments were to “Reach to the target as fast as possible when ready”. Speed of movement was prioritized over accuracy and participants were reminded to move quickly throughout data collection. The participant was positioned at the virtual display such that the Home position aligned with the sternum. Pupil distance and head width were used for calibration of the virtual display. A single electromagnetic marker was positioned on the nail bed of the right index finger and a black glove was donned. The glove, combined with darkening the room, served to block vision of the arm and hand throughout task performance eliminating visual feedback while reaching.

After a short exposure period to orient to the VE, a series of 24 practice trials were completed (12 with online visual feedback and 12 with post-response visual feedback only). Additional practice trials were provided if needed to confirm the participant understood the location of the targets and the trial sequence. Participants then performed a total of 168 reaching trials (7 blocks of 24 trials). Within each block, targets were presented in a pseudorandom order such that no consecutive trials were to the same target and each target was presented four times. After block 7, extra trials were collected if any errors occurred on individual trials. The first 2 blocks (48 trials) were dropped from data analysis to eliminate any effects of learning related to the VE; the remaining 120 trials (20 trials to each target) were used in analyses.

Following a short break of 20 to 30 minutes, participants completed the RWE condition. Participants completed a series of 12 practice trials to become familiar with the trial sequence; additional practice trials were provided as needed. Next, participants completed 120 trials (20 trials to each target) in 5 blocks of 24 trials each. Within each block, targets were presented in a pseudorandom order such that no consecutive trials were to the same target and each target was presented four times. After block 5, extra trials were collected if any errors occurred on individual trials.

### Kinematic data

The 3-D position of the index finger was collected through the electromagnetic marker at a sampling rate of 120 Hz throughout each reach trial. All kinematic data were filtered with a low-pass 2nd order Butterworth with a 10 Hz cut-off and differentiated to determine velocity and acceleration [34]. Movement onset was determined by searching backward in time from the peak of velocity until velocity dropped below 20 cm/sec and either changed direction or the change in velocity was less than 3 cm/sec for 2 consecutive samples, whichever was identified first. To eliminate any obvious corrections at the end of the movement, movement offset was determined by searching forward in time from the peak of velocity until velocity dropped below 20 cm/sec and either changed direction or the change in velocity was less than 0.3 cm/sec, whichever was identified first. All trials were visually inspected to confirm the accuracy of the movement onset and offset determination and manually corrected as necessary to eliminate corrections not caught by the automated algorithm. Trials where the movement time was >2 standard deviations longer than the mean for a given target were dropped from analysis to minimize any effect of movement speed on the control pattern utilized.

The following variables were extracted for each trial: movement distance, movement time, endpoint error, reach path ratio, peak velocity, peak acceleration, and time of peak velocity. Movement distance was the 3-D linear distance between the position at movement onset and the position at movement offset. Movement time was the time between movement onset and movement offset. Endpoint error was defined as the 3-D linear distance between the position at movement offset and target position. Reach path ratio, a measure of the straightness of the hand path, was calculated as the ratio of the actual path distance taken compared to the straight line distance between the position at movement onset and the position at movement offset. A ratio of 1 indicates a perfectly straight hand path while a ratio greater than 1 indicates curvature in the path taken to the target. Since we were interested in the planning of reach actions and whether early kinematic variables predicted eventual movement distance, we extracted the first peak of velocity and acceleration even if there were later additional peaks that were larger. Peak velocity was determined by searching forward in time from movement onset to the first peak of velocity that was followed by 2 consecutive frames where velocity decreased. Time of peak velocity was the time that corresponded to the initial peak of velocity. Peak acceleration was determined by searching forward in time from movement onset to the time of initial peak velocity for the first peak of acceleration that was followed by 2 consecutive frames where acceleration decreased.

### Dependent measures

The primary goal of this study was to determine how individuals utilized anticipatory planning and compensatory adjustments to control reach extent in the two environments. To understand the control pattern utilized, we looked at how early kinematic variables correlated with movement distance for each individual participant by target direction and reach environment [5]. We first determined whether peak velocity correlated with movement distance; a significant, positive correlation demonstrates a scaling of peak velocity. Planning was defined by the correlation of peak acceleration with movement distance; a positive, significant correlation equates to scaling of peak acceleration, or planning based, pulse-height control. If no significant correlation was found, planning was not used to control the reach action.

Two analyses were performed to quantify the use of compensatory adjustments. First, to determine if feedback-based adjustments were present prior to peak velocity, the relationship between time to peak velocity, a marker of acceleration duration, and movement distance was quantified. A significant, positive correlation of time to peak velocity with movement distance indicates a scaling of acceleration duration. Such a relationship indicates the use of pulse-width control.

**Figure 2:**
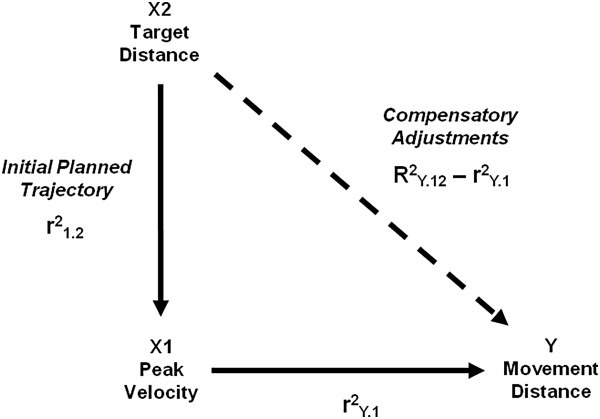
**Deterministic statistical model adapted from Gordon & Ghez (1987b).** In this model, target distance is hypothesized to influence movement distance (Y) through two pathways. In the initial planned trajectory, target distance influences movement distance through its effect on the initial peak of velocity (X_1_). The squared correlation coefficient *r*^*2*^_*Y.1*_ represents the percentage of variance in final movement distance explained by initial peak velocity. Through a second path, target distance (X_2_) influences the implementation of corrective adjustments to the trajectory to achieve the distance moved. The additional variance in movement distance accounted for by target distance is equal to the difference between the combined variance accounted for by initial peak velocity and target distance and the variance accounted for by peak velocity alone (*R*^*2*^_*Y1.2*_ - *r*^*2*^_*Y.1*_).

Next, to determine if additional compensatory adjustments were present after peak velocity that assisted in the achievement of eventual movement distance, a deterministic statistical model developed by Gordon & Ghez (1987b) was modified for determining the control of movement distance while reaching [23,32]. In this model, it is hypothesized that target distance (8, 16, 24 cm in the current experiment) has two independent effects on the control of reach extent (Figure 2). First, target distance is utilized to determine the initial trajectory of the reach and is reflected in the initial peak of velocity. The squared correlation coefficient describing the relationship between peak velocity and movement distance was defined by the following linear regression equation:

$$Y\;=\;a\;+\;b_{1}X_{1}$$

where Y = movement distance, X_1_ = initial peak velocity, a = Y-intercept, and b_1_ = regression coefficient expressing the change in Y for a given change in X_1_. Therefore, *r*^*2*^_*Y.1*_ reflects the percent variance in movement distance explained by variations in peak velocity and the degree to which movement distance is explained by the initial control pattern (pulse-height/pulse-width control).

Target distance also influences movement distance through a second, independent path (hatched line in Figure 2). This path represents the corrective influence of target distance on movement distance that acts to compensate for errors in the specification of peak velocity. To determine this effect, target distance is added to the above model yielding the regression equation:

$$Y\;=\;a\;+\;b_{1}X_{1}\;+\;b_{2}X_{2}$$

where Y = movement distance, X_1_ = initial peak velocity, *X*_2_ = target distance, a = Y-intercept, and b_1_ and b_2_ = regression coefficients. Therefore, *R*^*2*^_*Y.12*_ is the squared multiple correlation coefficient and reflects the percent variance in movement distance that can be explained by the linear combination of initial peak velocity and target distance. The additional variance in movement distance explained by target distance (compensatory adjustments) is equal to the difference in the proportion of variance explained by the combination of initial peak velocity and target distance and that accounted for by peak velocity alone (*R*^*2*^_*Y.12*_ – *r*^*2*^_*Y.1*_). If a statistically significant additional variance in movement distance is accounted for through this path, adjustments to the trajectory based on target distance are compensatory in nature; errors in the specification of peak velocity are corrected for to assist in the achievement of the actual distance moved.

### Statistical analysis

Correlation and statistical model analyses were carried out individually for each participant. Reaches were analyzed separately by target direction (+45°, -45°) secondary to differences in the magnitude of kinematic variables based on direction and to determine if the use of anticipatory planning and compensatory adjustments differed based on movement direction.

Comparison of the magnitude of kinematic variables (peak velocity, peak acceleration, movement time) between the VE and the RWE was performed using data from all reach trials for each individual participant with a paired *t*-test. All trials for all participants were then combined (20 trials x 6 targets x 5 participants) into a single analysis (paired *t*-test) to determine any group differences in kinematics between the two environments. For all correlation analyses, Pearson’s *r* is reported. The strength of relationships was interpreted based on the value of the correlation coefficient: *r* < 0.25 = little or no relationship; *r* of 0.25 to 0.50 = fair; *r* of 0.50 to 0.75 = moderate; *r* > 0.75 = strong [35]. Correlation coefficients for individual participants were transformed to a *z*-score (Fisher Z) and compared between target directions (+45°, -45°) and reach environments (VE, RWE) with a paired *t*-test. Mean *z*-scores were converted back to the equivalent correlation coefficients for reporting of results. The squared correlation coefficients from the deterministic statistical model were also compared with a paired *t*-test. Significance level was set at *p* < 0.05 for all statistical tests. SPSS 16.0 (SPSS,Inc., Chicago, IL) statistical software was used for all analyses.

## Results

### General characteristics of unconstrained reaching

**Figure 3:**
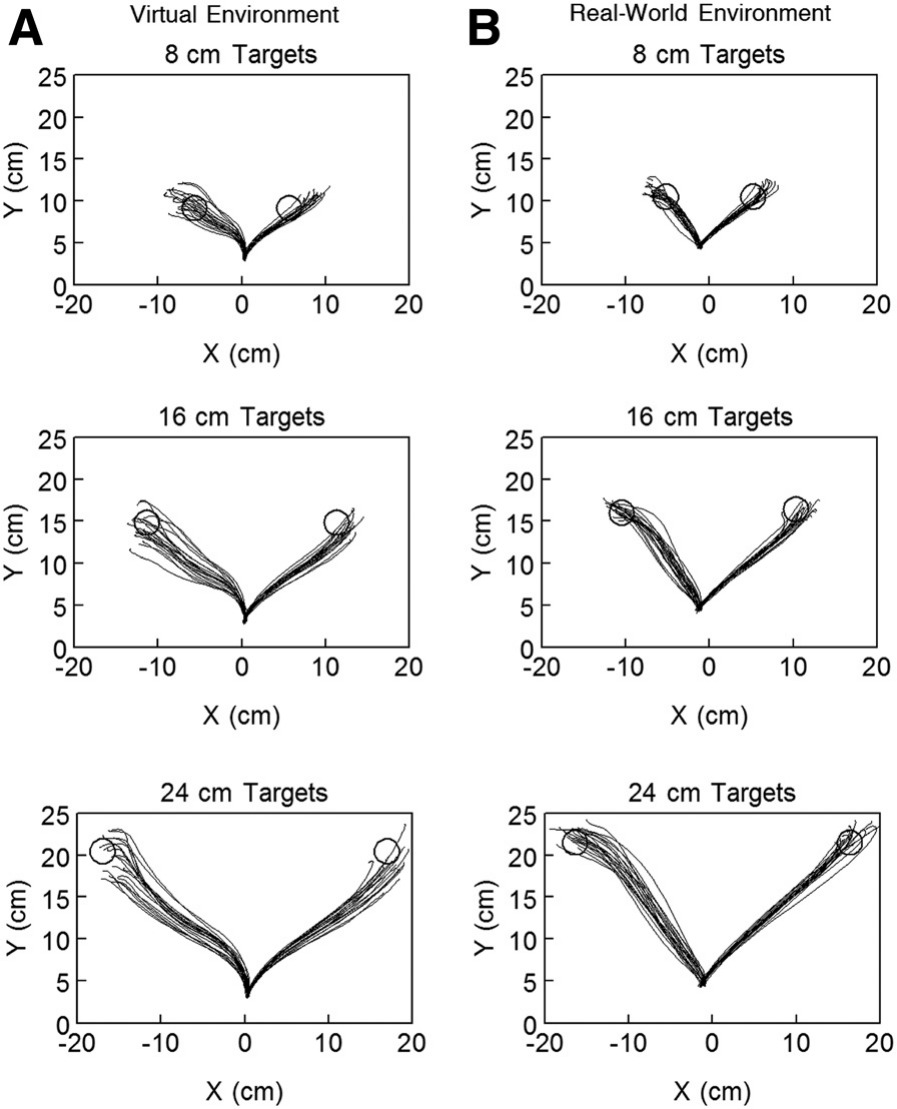
**Reach hand paths for one participant in both environments.** Reach hand paths for a single participant (S3) in the virtual environment (**A**) and the real-world environment (**B**) to the 8, 16, and 24 cm targets. Each line represents the hand path for a single reach trial. Open circle = target.

Figure 3 shows individual trial reach hand paths to each target in the VE and the RWE for a representative participant. In the VE, hand paths were directed to the target and relatively straight (mean hand path ratio: +45° targets = 1.06, -45° targets = 1.22). Reach hand paths in the RWE for this participant demonstrated characteristics similar to reaches in the VE (Figure 3) (mean hand path ratio: +45° targets = 1.07, -45° targets = 1.17).

**Figure 4:**
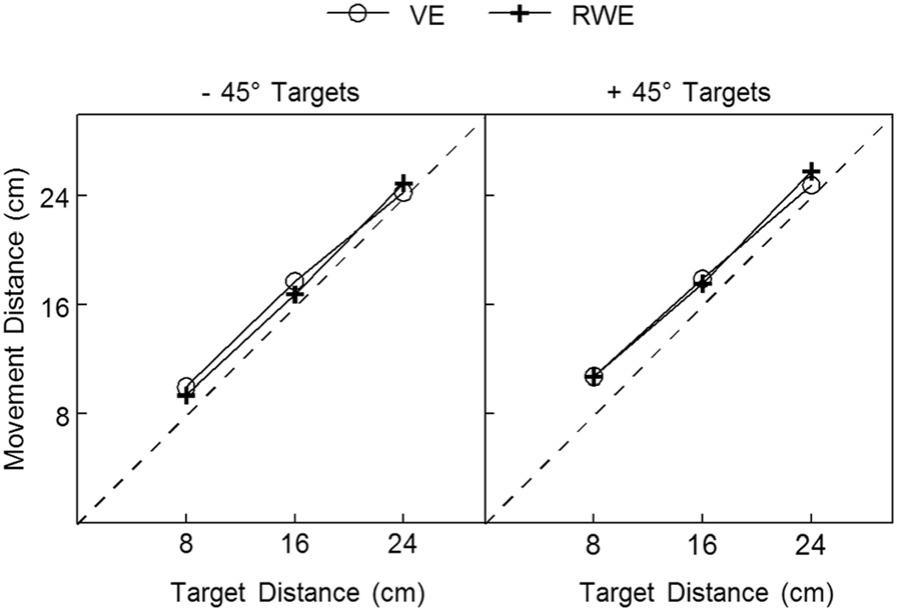
**Mean movement distance by reach direction in both environments.** Markers indicate group means with standard error bars. Dotted line indicates perfect scaling of movement distance to target distance. VE = virtual environment; RWE = real-world environment. Note that standard error was small across distances (<0.9 cm) and within the size of the markers.

As a group, movement distance scaled with target distance in both environments (Figure 4); movement distance was shorter for closer targets and longer for farther targets. Overall, participants tended to overshoot the target at all three distances in both the VE and the RWE. Hand paths demonstrated a similar degree of curvature in the two environments with greater curvature to the -45° targets (VE: 1.17 ± 0.02; RWE: 1.14 ± 0.03) than to the +45° targets (VE: 1.09 ± 0.02; RWE: 1.09 ± 0.02). Group endpoint error was slightly higher in the VE (2.6 ± 0.3 cm) compared with the RWE (1.8 ± 0.3 cm). However, post-response feedback when reaching in the VE allowed for up to 2.9 cm of error (based on the diameters of the target and the Cursor) for participants to receive feedback that they had ‘hit’ the target (target turned green). Therefore, mean error in the VE was within the resolution of the feedback provided to participants.

**Figure 5:**
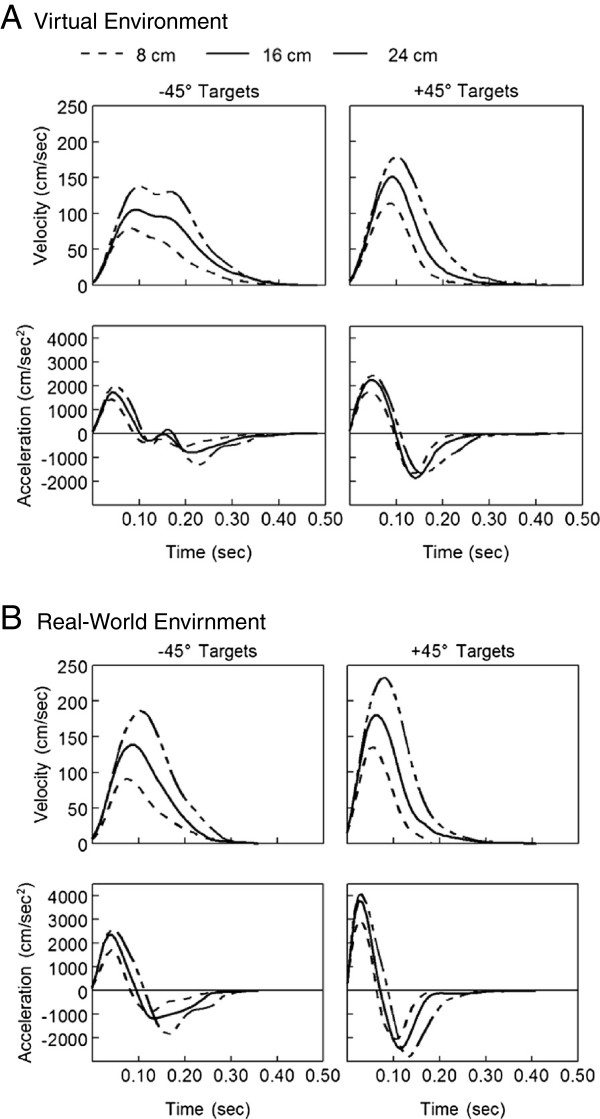
**Mean velocity and acceleration trajectories for one participant reaching in both environments.** Mean velocity and acceleration profiles are shown for the virtual environment (**A**) and real-world environment (**B**). Each line represents an ensemble average of all trials to a specific target distance (8, 16, 24 cm) for the same participant shown in Figure 3.

**Figure 6:**
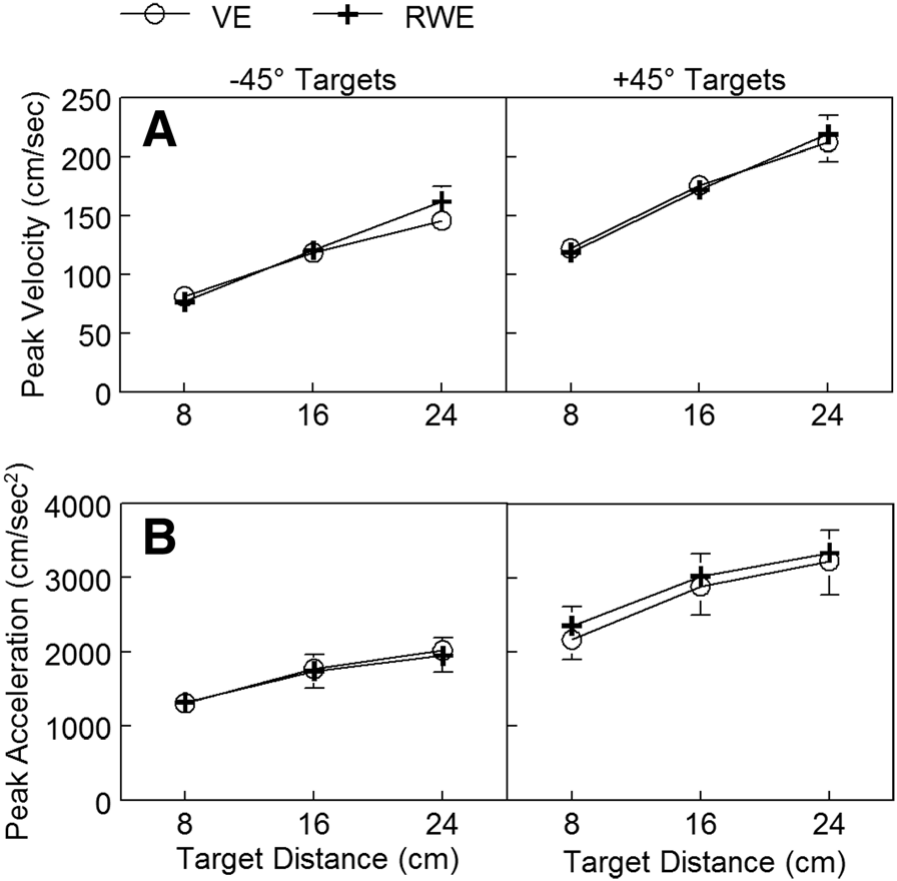
**Mean peak velocity and peak acceleration by reach direction in both environments.** Markers indicate group means with standard errors (down for VE, up for RWE) for each target distance for peak velocity (**A**) and peak acceleration (**B**). VE = virtual environment; RWE = real-world environment.

Velocity and acceleration were not always single-peaked as has been commonly reported for reach actions under more constrained conditions [23,24,36]. Additional peaks in velocity were generally due to curvature of the hand path related to the unconstrained nature of the task. Ensemble average velocity and acceleration for the same participant in Figure 3 are shown in Figure 5 for the VE and RWE. In both environments, peak velocity scaled with target distance such that reaches to the 8 cm targets had a lower average peak velocity than reaches to the 24 cm targets. Peak acceleration also scaled with target distance although this scaling was less distinct. While peak acceleration on average was higher for farther targets compared with closer targets, there was more variability and therefore more overlap in peak acceleration between targets compared to peak velocity. This scaling of peak velocity and peak acceleration to target distance was consistent across participants (Figure 6).

While movements were overall relatively quick (average < 400 msec), movements times were longer with lower peaks of acceleration and velocity for reaches to the -45° targets compared with reaches to the + 45 targets in both the VE and RWE (Figure 6, Table 1). These differences are consistent with changes in reach kinematics due to the effects of inertia of the arm [22]. Reaches to the +45° targets required primarily elbow extension while reaches to the -45° targets involved reaching across midline and required both elbow extension and shoulder flexion.

**Table 1:** Comparison of reach kinematics between environments

	**Direction**	**N**	**Peak velocity (cm/sec)**			**Peak acceleration (cm/sec**^**2**^**)**			**Movement Time (sec)**		
			**VE**	**RWE**	***p*-value**	**VE**	**RWE**	***p*-value**	**VE**	**RWE**	***p*-value**
**S2**	+45°	60	191.75 ± 45.89	149.61 ± 41.01	<0.001*	3935.09 ± 905.79	2528.42 ± 640.39	<0.001*	0.198 ± 0.055	0.235 ± 0.047	<0.001*
	−45°	59	131.08 ± 34.10	105.95 ± 35.67	<0.001*	2424.23 ± 834.51	1475.10 ± 408.14	<0.001*	0.315 ± 0.078	0.355 ± 0.058	<0.001*
**S3**	+45°	60	150.37 ± 34.15	184.65 ± 42.63	<0.001*	2181.49 ± 521.17	3733.70 ± 635.66	<0.001*	0.276 ± 0.080	0.230 ± 0.074	<0.001*
	−45°	60	110.46 ± 27.36	140.06 ± 41.57	<0.001*	1762.22 ± 421.91	2307.15 ± 538.38	<0.001*	0.378 ± 0.053	0.276 ± 0.042	<0.001*
**S4**	+45°	60	137.46 ± 32.38	138.96 ± 34.80	0.602	1950.38 ± 401.73	2260.18 ± 534.05	<0.001*	0.275 ± 0.054	0.245 ± 0.067	<0.001*
	−45°	60	100.54 ± 26.59	89.44 ± 26.83	<0.001*	1136.05 ± 213.28	1188.34 ± 249.58	0.115	0.386 ± 0.052	0.364 ± 0.053	<0.001*
**S5**	+45°	60	176.24 ± 50.23	176.46 ± 50.83	0.961	2460.29 ± 704.88	2591.62 ± 745.17	0.169	0.267 ± 0.075	0.218 ± 0.044	<0.001*
	−45°	58	109.49 ± 29.27	123.33 ± 41.66	<0.001*	1306.79 ± 363.51	1439.23 ± 481.84	0.031*	0.366 ± 0.066	0.330 ± 0.047	<0.001*
**S6**	+45°	60	193.62 ± 54.88	199.54 ± 56.15	0.125	3278.70 ± 798.53	3426.06 ± 798.53	0.161	0.199 ± 0.036	0.209 ± 0.056	0.143
	−45°	60	123.72 ± 35.52	138.26 ± 40.74	<0.001*	1846.80 ± 543.41	1933.76 ± 476.45	0.145	0.291 ± 0.057	0.284 ± 0.057	0.385
**Group**	+45°	300	169.89 ± 49.49	169.84 ± 50.64	0.984	2761.19 ± 1009.45	2908.00 ± 862.85	0.033*	0.235 ± 0.072	0.227 ± 0.060	0.089
	−45°	297	115.04 ± 32.47	119.53 ± 42.21	0.009*	1695.38 ± 685.39	1670.96 ± 595.12	0.570	0.339 ± 0.072	0.323 ± 0.063	<0.001*

*N* = Number of reach trials; *VE* = Virtual Environment; *RWE* = Real-World Environment.

* Significant differences.

### Comparison of kinematic variables between VE and RWE

Previous studies have reported differences in the magnitude of some kinematic variables when comparing reach actions performed in VR to reach actions in the real-world [10,11]. While participants achieved a scaling of movement distance in both the VE and RWE in the current experiment, some differences in the absolute value of kinematic variables were found. Table 1 lists the average peak velocity, peak acceleration, and movement time for each participant as well as the overall group mean. Significant differences for individual participants were found in 7/10 comparisons for peak velocity, 6/10 for peak acceleration, and 8/10 for movement time. In 3 of the 5 participants, when significant differences in kinematics were found, participants tended to have higher peak velocity, higher peak acceleration, and shorter movement times when reaching in the RWE compared with the VE. S2 demonstrated the opposite pattern; peak velocity and peak acceleration were lower and movement time longer in the RWE. S6 showed very little difference in all variables between the two reach environments. For the group, some significant differences in kinematic variables were found, however, the magnitude of the differences were quite small (e.g. 4.5 cm/sec for peak velocity to -45° targets) and were therefore not likely to impact the primary analyses of interest.

### Anticipatory planning for reach actions

**Figure 7:**
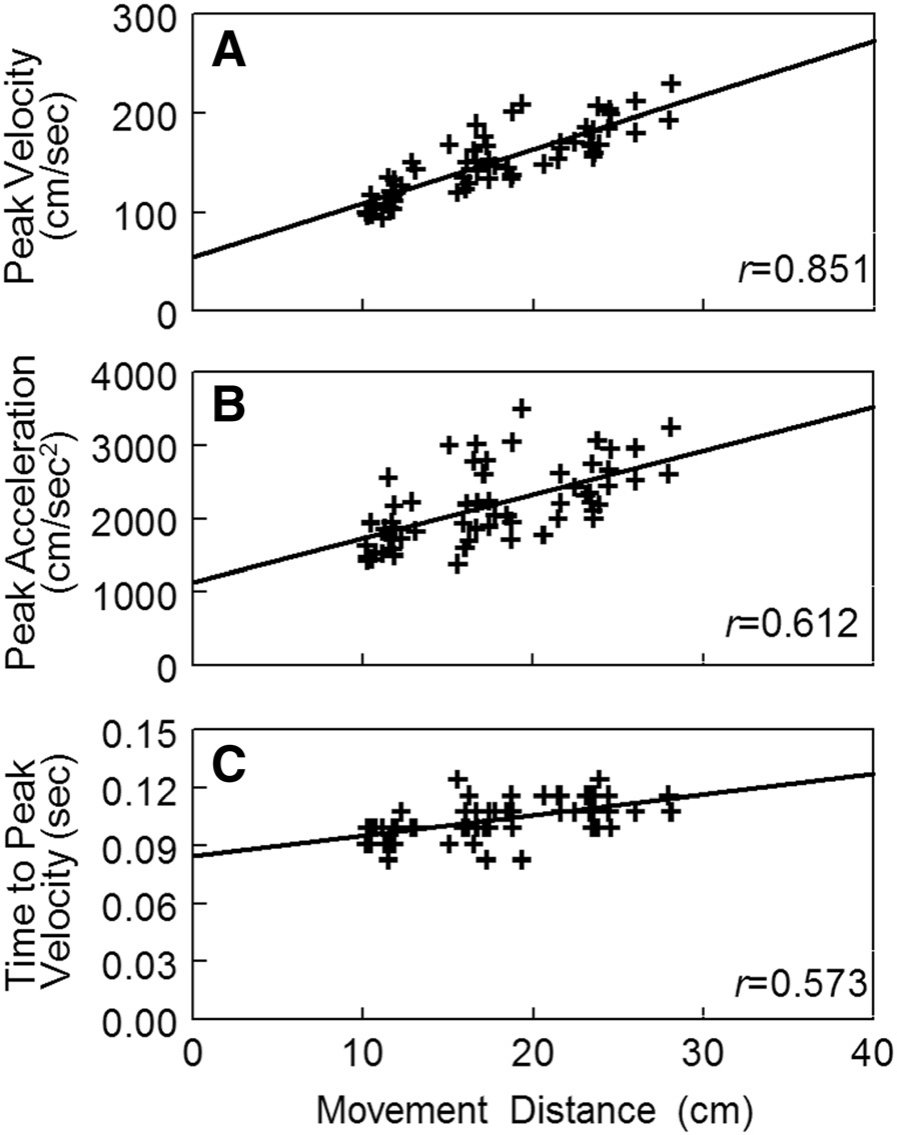
**Peak velocity (A), peak acceleration (B), and time to peak velocity (C) by movement distance.** Data presented for a single participant (S3) reaching to the +45° targets in the virtual environment. Each data point represents a single reach trial. Solid line indicates the linear regression line fit to the data. *r* = correlation coefficient between movement distance and the kinematic variable (peak velocity, peak acceleration, time to peak velocity). All correlations significant at *p* < .01.

**Figure 8:**
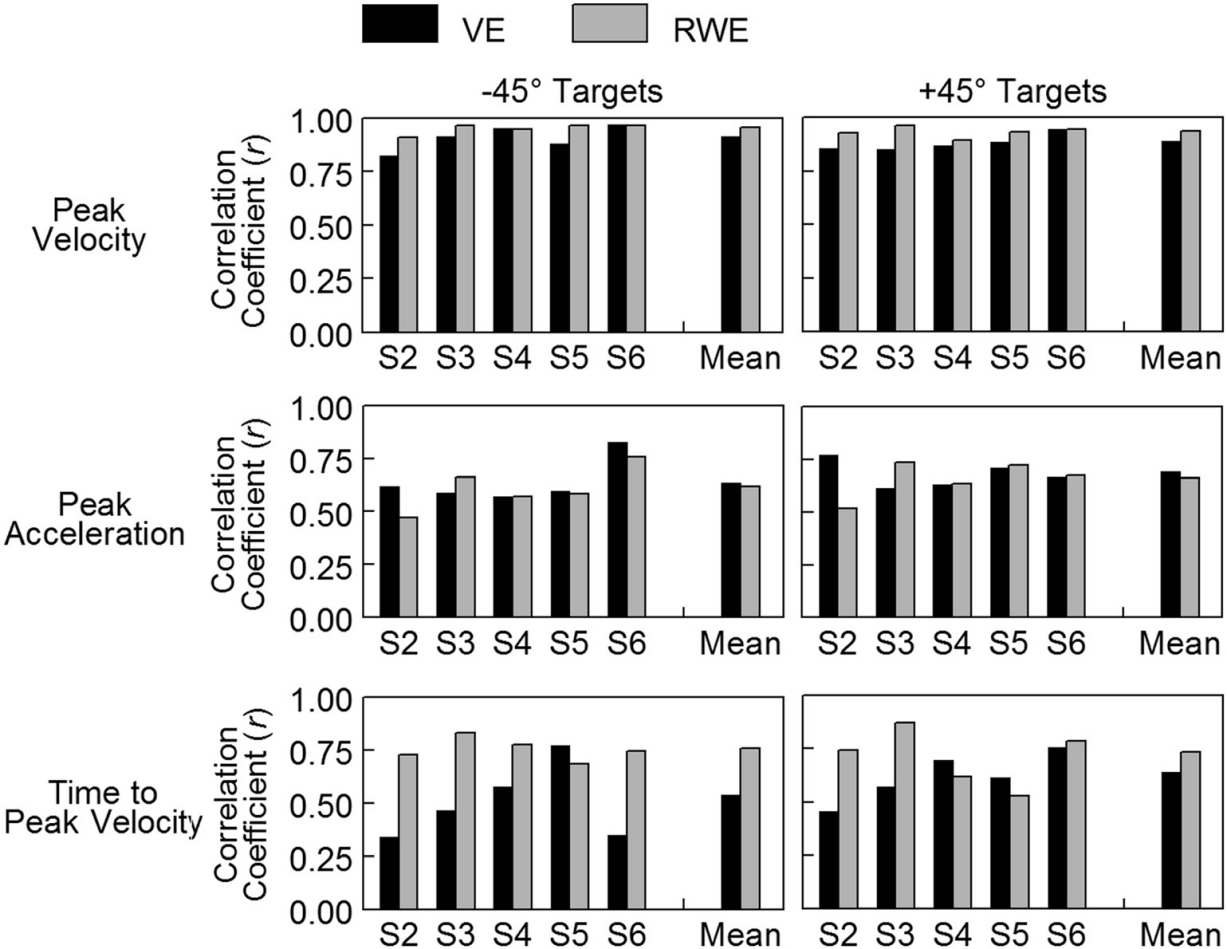
**Correlation of peak velocity, peak acceleration, and time to peak velocity with movement distance.** Correlation coefficient *r* shown for each participant in the virtual environment (VE) and the real-world environment (RWE) reaching in both directions (+45°, −45°). Mean represents the average across all participants calculated with the transformed Fishers *z* scores.

We examined the degree to which several kinematic markers correlated with movement distance to determine whether reach extent was specified early in the movement. Figure 7A shows the correlation of peak velocity with movement distance for a single representative participant for reaches to the +45° targets in the VE. For movements of shorter distances, the participant demonstrated a lower peak velocity relative to movements of longer distances. This strong and significant correlation between peak velocity and movement distance was consistent across all 5 participants reaching in the VE with correlation coefficients ranging from 0.825 to 0.969 (Figure 8). The correlation of peak velocity with movement distance in the RWE, though slightly higher, was not significantly different from the VE (+45°: *p* = .066; −45°: *p* = .109). Additionally, there was no difference in the correlation of peak velocity with movement distance based on target direction in either environment (VE: *p* = .280; RWE: *p* = .188).

To determine whether this scaling of peak velocity was preplanned, we examined the correlation of peak acceleration with movement distance. Peak acceleration occurred on average 51 msec in the VE and 37 msec in the RWE after movement onset and, therefore, represented the early component of the movement prior to the availability of feedback. Figure 7B shows the correlation of peak acceleration with movement distance for a single participant reaching in the VE. Similar to peak velocity, lower peaks of acceleration tended to correspond to shorter movement distances while higher peaks of acceleration tended to correspond to longer movements although the correlation was lower than peak velocity. Peak acceleration had a significant and moderate correlation with movement distance in all participants for both directions in the VE (range 0.515 to 0.825) and the RWE (range 0.474 to 0.756) (Figure 8) suggesting individuals were utilizing pulse-height control. There was no significant difference in correlation values based on target direction (VE: *p* = .368; RWE: *p* = .336) or reach environment (+45°: *p* = .773; -45°: *p* = .392). The magnitude of the correlations of peak acceleration with movement distance found here are similar to previous reports for reaches under a more constrained condition [5].

### Compensatory adjustments to reach actions prior to peak velocity

While peak acceleration was a moderate to strong predictor of movement distance, there was some variability between subjects (*r*^2^ ranged from 25.2% to 67.4% across the two environments). Participants did use pulse-height control that relied on planning to initiate the movements although a fair amount of variance in movement distance was not accounted for by peak acceleration. After the time of peak acceleration, feedback is available to adjust the duration of the initial acceleration pulse to maximize goal achievement [30,37]. In the current task, visual feedback was not provided during reaching; any feedback based adjustments had to have been made based on internally derived proprioceptive information [30]. Since the duration of the initial acceleration pulse corresponds to the time of initial peak velocity, we calculated the correlation between the time to peak velocity and movement distance to determine if individuals scaled the duration of acceleration and utilized pulse-width control [5].

The relationship between time to peak velocity and movement distance for a single participant is shown in Figure 7C. Time to peak velocity had a significant, moderate correlation with movement distance suggesting this participant used pulse-width control in addition to pulse-height control to achieve the actual distance moved. This pattern was consistent across all 5 participants in the VE and the RWE (Figure 8). There was no significant difference in the correlation of time to peak velocity with movement distance based on target direction (VE: *p* = .238; RWE: *p* = .606). The correlation of time to peak velocity with movement distance tended to be higher in the RWE in both target directions but this difference did not quite reach statistical significance (+45°: *p* = .299; −45°: *p* = .059). Therefore, participants utilized a combined pulse-height, anticipatory planning strategy in combination with a pulse-width, feedback adjustment based strategy to control reach extent in both environments.

**Figure 9:**
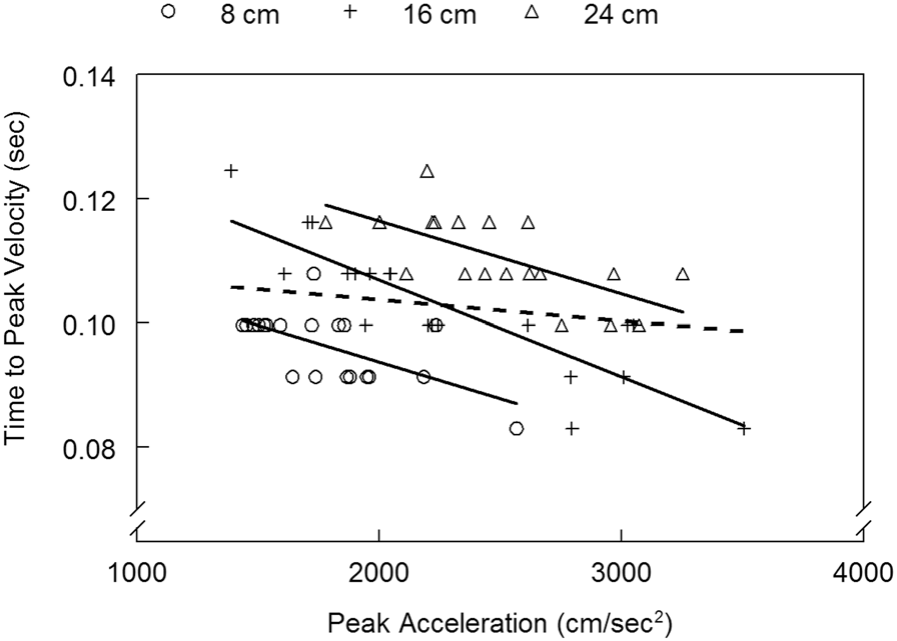
**Relationship between time to peak velocity and peak acceleration by target distance.** Data is from the same participant shown in Figure 3 reaching to the +45° targets in the virtual environment. Data points represent individual reach trials to each target (circle = 8 cm target; cross = 16 cm target; triangle = 24 cm target). Solid lines are the linear regression lines fit to the data for each target (correlation coefficients: *r* = -0.616 for 8 cm target; *r* = -0.856 for 16 cm target; *r* = -0.680 for 24 cm target; all significant at *p* < 0.01). Dotted line is the linear regression line fit to all data points combined across targets (*r* = -0.179, *p* = .172).

Pulse-width control is hypothesized to utilize feedback for correction of errors in the initial specification of acceleration [30,31]. If acceleration duration is adjusted based on initial errors in peak acceleration, there should be a significant relationship between peak acceleration and time to peak velocity. Figure 9 shows this relationship for a single participant reaching to the +45° targets in the VE. There was no significant correlation between peak acceleration and time to peak velocity when reaches to all 3 targets were included (*r* = -0.179, *p* = 0.172). There was a moderate to strong and significant negative correlation between peak acceleration and time to peak velocity when only reaches to a single target were considered (8 cm, *r* = -0.616; 16 cm, *r* = -0.856; 24 cm, *r* = -0.680; all *p* < .01). For example, when peak acceleration was lower to the 8 cm target, time to peak velocity, or acceleration duration, was relatively prolonged; when peak acceleration was higher to the 8 cm target, time to peak velocity was relatively shorter. Therefore, the duration of the acceleration pulse (time to peak velocity) was adjusted to correct for errors in the initial specification of acceleration magnitude, e.g. if peak acceleration was relatively high for a given target, the duration of the acceleration pulse was shortened in order to achieve the desired trajectory. This control pattern was consistent across participants and environments. In the VE, 25 out of 30 correlations (5 participants x 6 targets) were significant and negative while in the RWE, 28 out of 30 correlations were significant and negative.

### Compensatory adjustments to reach actions after peak velocity

**Figure 10:**
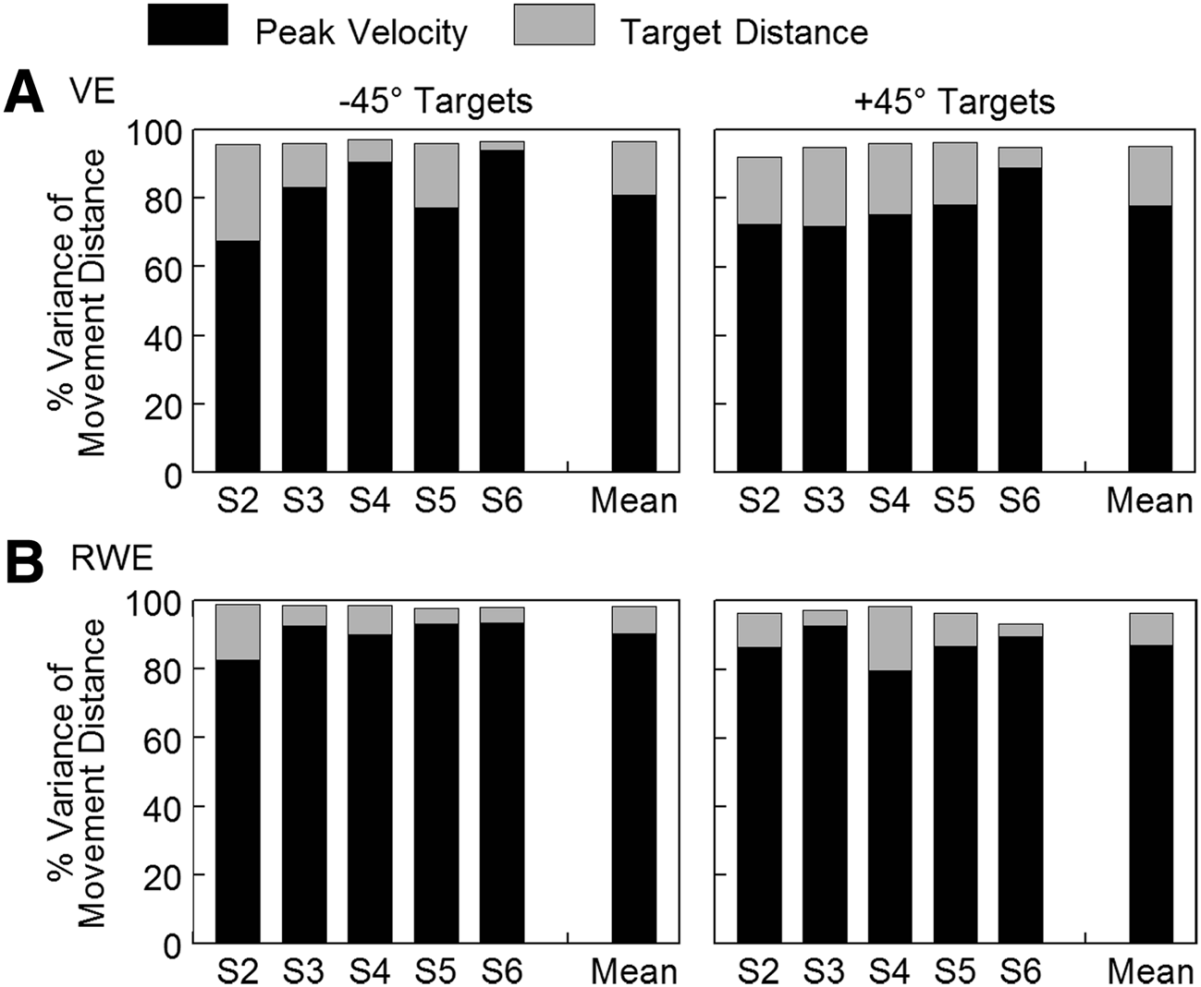
**Percent variance in movement distance accounted for by peak velocity and target distance.** Data shown for each individual participant reaching in both directions in the virtual environment (VE) (**A**) and the real-world environment (RWE) (**B**). Group means are at the right of each plot. The height of the black portion of the bar represents the variance in movement distance explained by peak velocity. The top portion of the bar represents the additional variance in movement distance explained when target distance is added to the regression model (see Figure 2). The overall height of the bar represents the total variance accounted for by the two factors combined (peak velocity, target distance).

Peak velocity was a strong predictor of movement distance across participants with *r*^2^ ranging from 67.4% to 93.8% in the VE and 79.6% to 93.5% in the RWE (Figure 10). While peak velocity predicted a significant amount of the variance in movement distance, the remaining variance unaccounted for ranged from 6.2% to 32.6% across participants. Using a deterministic statistical model (Figure 2), we examined whether additional variance in movement distance was accounted for by adding target distance to peak velocity. If target distance added significantly to the variance in movement distance accounted for, adjustments to the movement trajectory were made by participants based on target information that were effective in assisting achievement of the eventual distance moved.

In the VE, the explained variance in movement distance increased significantly when target distance was added to model; the added variance ranged from 6% to 28.2% across participants (Figure 10A). The total variance accounted for by the 2 variables (peak velocity, target distance) was >92% in all participants. Similar results were found for reaches in the RWE. An additional 4.4% to 18.7% of the variance in movement distance was explained when target distance was added to the model (Figure 10B). The total variance accounted for by the regression model in the RWE was >93% across participants. Therefore, the independent influence of target distance added significantly to the variance accounted for in movement distance in both environments.

Target direction had no effect on the variance explained by peak velocity, compensatory adjustments, or the overall model in the VE or the RWE (*p* > 0.05). The percent variance of movement distance explained by peak velocity tended to be higher in the RWE compared to the VE but this difference did not reach statistical significance (+45° targets: *p* = 0.052; -45° targets: *p* = 0.092). The added variance accounted for by adding target distance to the model (compensatory adjustments) was lower in the RWE compared to the VE but again this difference did not quite reach statistical significance for either direction (+45° targets *p* = 0.051; -45° targets: *p* = 0.158). The total variance in movement distance accounted for by both factors was slightly higher in the RWE; this difference was statistically significant for reaches to the -45° targets only (*p* < 0.01) although the actual differences between the means was small (2.1%). Mean values for both environments and directions were >95% suggesting that the model predicted movement distance similarly in the two reach environments.

## Discussion

When reaching in an immersive VE, reach behavior demonstrated similar characteristics to behavior from other studies: relatively straight hand paths and systematic scaling of peak velocity based on target distance [5,22-24,26,36]. Movement time, peak velocity, and peak acceleration were lower when reaching across midline (−45° targets) compared to reaching ipsilaterally (+45° targets) suggesting that the influence of inertia on reach behavior [22] was similar in an immersive VE as it is in other conditions. Some differences in movement execution kinematic variables were found between environments but these differences varied between individual participants and did not impact the overall use of anticipatory planning and compensatory adjustments in the control of movement distance.

To our knowledge, this is the first study to directly assess the impact of a VE on the planning and adjustments of reach movements to targets that vary in distance. Clarification of the influence of a VE on the planning of goal-directed actions is an important step in validating VR for the investigation of motor control related research questions due to the known impact of environmental cues on motor planning [13-16]. Our results suggest that the use of initial planning and compensatory adjustments to control reach extent is similar in an immersive VE compared to an analogous RWE. Overall, previous research has shown that the execution of reach movements in VR have similar kinematic features to reach movements in the real-world [9,11,38] although some differences have been reported in older adults reaching with a head-mount display [11,38] or when a grasp requirement is added [10,12]. However, none of these studies addressed whether the VE impacted the planning of UE reach actions.

In both the VE and the RWE, participants utilized a combined planning based, pulse-height/feedback based, pulse-width control pattern to achieve a scaling of peak velocity to the actual distance moved. This control pattern differs slightly from that reported by Sainburg & Schaefer (2004) for goal-directed reaches that required a single-joint of motion, elbow extension, in right-hand dominant individuals. In that study, individuals relied more on a pulse-height control pattern than a pulse-width control pattern with the right arm when reaching to target presented in the ipsilateral workspace. In our study, reaches were unconstrained and required multi-joint movement. The increased use of pulse-width control in our participants may be related to the increased inertial load of the arm [26] imposed by the unconstrained nature of the task that required holding the arm against gravity. This added load may have served to decrease the influence of the initial plan on the eventual movement outcome.

While individuals in our study did utilize initial planning, peak acceleration only predicted approximately 25% to 67% of the variance in movement distance, overall less than that reported by Messier & Kalaska (1999) for reaches to two-dimensional (2-D) targets. Yet, individuals were able to make effective adjustments to acceleration duration based on the variability in the specification of the initial plan [30,39] early in the movement. Such a control pattern is consistent with the idea of an internal model for the control of reach actions [40,41]. With an internal model, once the initial plan is transformed to a motor output, it is hypothesized that an efferent copy of that command is sent to a forward model. The forward model predicts the response of the arm to the given motor output allowing the ability to monitor for errors and adjust performance early after movement onset. Such a system does not require a unique plan for each reach action but instead allows for a more general plan that can be adapted as needed based on environmental conditions and system noise leading to greater overall movement flexibility.

Additional compensatory adjustments were present after the time of peak velocity consistent with previous reports [23,32]. In fact, the total variance in movement distance accounted for by the two factor statistical model was >92% in all participants. Therefore, we describe here an evolving control strategy for reach actions to targets that vary in distance. Individuals initiated the reach with an initial plan that provided a ‘ball-park’ start to achieve the desired movement distance. Early adjustments to acceleration duration were made based on initial variability in the plan, possibly through information provided by a forward model, that assisted in the achievement of the eventual distance movement. Further compensatory adjustments to the trajectory were made after the time of peak velocity based on target information that assisted in the achievement of the distance moved.

There are some limitations to consider when interpreting the results of the current study. First, while the RWE environment used in this experiment was similar to the VE, it was not possible to fully replicate the environment presented in the VR system. The RWE was similar to the VE as to spatial target location, unavailability of visual feedback of hand position during reaching, ending the reach in free space (no haptic feedback), and provision of post-response visual feedback of finger position in relation to target location. The RWE differed from the VE as to the mode of target goal presentation (verbally in the RWE, visually in the VE) and visualization of the target during reaching (not visible in the RWE as eyes were closed, visible in VE as eyes were open). Although reaching to remembered targets compared to visual targets can affect movement planning [18,19], this difference did not impact the behavior described here consistent with a similar experimental set-up in Messier & Kalaska (1997). Second, the findings of the current study may be specific to the VR display system used in this experiment, specifically an immersive VE where objects are presented in a first-person perspective. Other VEs that utilize a computer screen for target presentation, include only a 2-D workspace, or use video capture to project the user into the environment may have different sensorimotor transformation requirements and, therefore, different effects on motor planning than those reported here. Third, the small sample size in this study may not have provided enough power to find true differences in execution and planning variables between the VE and RWE. However, the overall control pattern used to control reach extent was consistent across participants and environments: an initial plan followed by compensatory adjustments before peak velocity and compensatory adjustments after peak velocity. While a larger sample may find differences in the magnitude of variables, the current data suggest that the overall control pattern would likely persist. Finally, the order of reach environment was the same across participants, VE followed by the RWE. Reaches in the VE may have had an influence on reaches in the RWE that could have impacted the results.

## Conclusion

An immersive, VE is a valid environment for the investigation of the use of initial planning and compensatory adjustments for unconstrained reach actions. Findings in such an environment can be used to understand the control of goal-directed aiming in real-world contexts. Future experiments that include additional manipulations that take advantage of the possibilities provided by VR can be performed without the need to recreate an analogous real-world task for comparison.

## Competing interests

The authors declare that they have no competing interests.

## Authors’ contributions

JCS conceived of the study, designed the experiment, performed data collection and data analysis, and wrote the manuscript. JG participated in study design, data analysis, and data synthesis. CJW participated in study design, data analysis, data synthesis, and manuscript preparation. All authors read and approved the final manuscript.
